# Genetic characterization of *Fasciola hepatica* (Linnaeus, 1758) in cattle from Paraná, Brazil

**DOI:** 10.1590/S1984-29612025069

**Published:** 2025-12-05

**Authors:** Desiree Vera Pontarolo, Gilberto Augusto Krug, Pedro Mesquita Fonseca, Daniel Angelo Sganzerla Graichen, Marcelo Beltrão Molento

**Affiliations:** 1 Universidade Federal do Paraná – UFPR, Departamento de Medicina Veterinária, Curitiba, PR, Brasil; 2 Universidade Federal de Santa Maria – UFSM, Departamento de Biologia, Palmeira das Missões, RS, Brasil

**Keywords:** Fasciolosis, gene flow, genetic variability, zoonosis, phylogenetics, liver condemnation, Fasciolose, variação genética, fluxo genético, zoonose, filogenética, descarte de fígado

## Abstract

Fasciolosis is a foodborne anthropozoonotic disease caused by *Fasciola* spp. that affects multiple hosts worldwide. Genetic characterization studies have revealed considerable diversity within *F. hepatica* populations owing to human and animal migration and intermediate snail hosts. Molecular markers such as microsatellites and mitochondrial DNA sequences are useful tools for assessing parasite population dynamics and evolutionary history. In this study, 16 *F. hepatica* samples were analyzed using cytochrome c oxidase 1 (*CO1*) and nicotinamide dehydrogenase (*NADH*) genes. Parasites were collected from the livers of naturally infected cattle originating from 14 municipalities in Paraná, Brazil. Both CO1 and NADH sequences showed high homogeneity, with mean genetic distances <1% (0.0084 and 0.0092, respectively). Network analyses revealed seven CO1 haplotypes and four NADH haplotypes among the new Paraná samples. When compared with reference sequences available in GenBank from other Brazilian states (Rio Grande do Sul and São Paulo), the Paraná samples also displayed <1% genetic divergence. This high level of homogeneity suggests a common and stable origin, with limited gene flow across regions. The genetic information reported here can support the development of targeted interventions, such as vaccines and drugs, aimed at controlling similar *F. hepatica* genetic variants.

## Resumo

A fasciolose é uma antropozoonose transmitida por alimentos causada por *Fasciola* spp., que infecta múltiplos hospedeiros. A diversidade genética de *F. hepatica* resulta da migração humana e animal, da presença de hospedeiros intermediários e do uso da terra. Marcadores moleculares, como microssatélites e DNA mitocondrial, permitem estudar sua dinâmica populacional e história evolutiva. Neste estudo, 16 amostras de *F. hepatica* foram analisadas utilizando os genes citocromo c oxidase 1 (CO1) e nicotinamida desidrogenase (NADH). Os parasitos foram coletados a partir de fígados de bovinos naturalmente infectados, provenientes de 14 municípios do Paraná, Brasil. Tanto as sequências de CO1 quanto de NADH apresentaram alta homogeneidade, com distâncias genéticas médias <1% (0,0084 e 0,0092, respectivamente). As análises de rede revelaram sete haplótipos de CO1 e quatro haplótipos de NADH entre as novas amostras do Paraná. Quando comparadas com sequências de referência disponíveis no GenBank de outros estados brasileiros (Rio Grande do Sul e São Paulo), as amostras do Paraná também apresentaram divergência genética <1%. Esse alto grau de homogeneidade indica origem comum e fluxo gênico limitado entre regiões. Os dados genéticos podem subsidiar o desenvolvimento de vacinas e fármacos para o controle de variantes semelhantes de *F. hepatica*.

## Introduction

Fasciolosis is a neglected anthropozoonotic disease that affects food safety (Molento et al., 2018). The infection is caused by two liver fluke species (*Fasciola hepatica* and *F. gigantica*), where 2.4 million people are infected in > 70 countries, with a significant financial impact (Arias-Pacheco et al., 2020; Davis et al., 2020). This disease affects various ruminants (such as cattle and goats), and pigs, horses, and humans (Howell et al., 2020; Nyindo & Lukambagire, 2015). Moreover, evidence indicates that fasciolosis continues to be more prevalent in Brazil's southern and southeastern regions, with occasional reports from other parts of the country (Almeida et al., 2024). The prevalence of fasciolosis has recently increased because of alterations in climatic conditions (Afshan et al., 2014; van Dijk et al., 2010), as well as changes in land use and cover (Almeida et al., 2024). The rate of liver infection in the southern region of Brazil has increased by 30%, with specific areas recording rates > 70% (Bennema et al., 2014; Martins & Sperandio, 2024). Projections indicate that this trend will persist well into the foreseeable future (Fox et al., 2011; Molento et al., 2020).

*Fasciola hepatica* is a liver fluke believed to have recolonized Europe during the last Ice Age. Genetic evidence, including the β-tubulin 3 gene and Asian mitochondrial lineages, supports this theory. These migrations are likely linked to human and animal movements from Asia to Europe (Semyenova et al., 2006; Teofanova et al., 2011). The global spread of fasciolosis is extensive, stemming from pre-historical migration in the late Holocene (Beltrame et al., 2017) to old-world livestock during colonization periods. This is facilitated by the vast distribution of Lymnaeidae snails, which serve as intermediate hosts for the parasite (Kasahara et al., 2021). Although *F. hepatica* was associated with the European colonization of the Americas 400 years ago, Beltrame et al. (2020) found trematode eggs in deer coprolites in Argentina with mtDNA *CO1* and *NADH* genes compatible with *F. hepatica,* suggesting a much earlier presence.

Accurate identification of *Fasciola* species based solely on morphological features remains problematic, primarily due to host-induced variations in parasite size, shape, and reproductive characteristics (Periago et al., 2006). Experimental studies using Wistar rats infected with *F. hepatica* from different definitive hosts have demonstrated that host species can significantly influence the morphology of adult flukes and their eggs (Periago et al., 2006; Valero et al., 2001). These limitations highlight the need for complementary molecular approaches that can provide more reliable and objective means of species identification. In recent years, mitochondrial markers such as cytochrome c oxidase subunit 1 (*CO1*) and NADH dehydrogenase subunit 1 (*NAD1*), as well as nuclear markers including 28S rRNA and internal transcribed spacers (ITS1 and ITS2), have been widely adopted as effective tools for distinguishing between closely related species and detecting hybrid forms of *F. hepatica* and *F. gigantica* (Ichikawa-Seki et al., 2017; Itagaki et al., 2011). These molecular techniques not only enhance the accuracy of species identification but also contribute to a better understanding of parasite population structure, transmission dynamics, and geographical distribution (Alkahtani et al., 2024; Alsulami et al., 2023; Kasahara et al., 2021). Nevertheless, their application is still limited in some regions due to restricted access to molecular facilities and incomplete reference databases, rather than inherent inefficiency of the techniques themselves.

Determining the level of population homogeneity using network analyses and nucleotide divergence (*CO1* and *NADH*) may reveal essential traits of habitat adaptation and shared environmental influences when comparing samples from different origins. Genetic diversity is a vital indicator of the evolutionary capacity of a species. The higher the level of gene diversity, the greater the adaptability to the environment by a species or population (Xifeng et al., 2022). Vázquez et al. (2016) verified the genetic diversity of *F. hepatica* using samples from nine cattle and water buffalo populations in the central-western region of Cuba. The authors found an essential number of polymorphisms at these four loci. However, no apparent genetic differences related to the area or host were found, suggesting a high rate of cross-fertilization between*F. hepatica* populations. One study in Brazil analyzed *F. hepatica* from Rio Grande do Sul (n = 69) and Paraná (n = 10) using *CO1* and *NADH* genes (Schwantes et al., 2020). Ten and 24 haplotypes were identified for *CO1* and *NADH*, respectively. The most represented haplotypes for both *CO1* and *NADH* in this study were shared with samples from at least nine countries, as previously recorded in GenBank. In contrast, 15 haplotypes were exclusively identified in both states and were exclusive to Brazil.

Although we have previously established the historical origin and geographical movement of *Fasciola*, further studies should focus on determining *F. hepatica* population constancy and gene flow under farming conditions. This study aimed to examine the genetic variability of adult *F. hepatica* parasites in 14 populations in Paraná, southern Brazil.

## Material and Methods

### Description of study area

Paraná state ranks second in national milk production and is recognized for its high productivity and value in the dairy sector (SEAB, 2024). The state has sufficient water reserves with two climate types (Cfa: subtropical humid and Cfb: tropical oceanic) based on the Köppen classification (Alvares et al., 2013). The average temperature is > 20 oC, and the rainy season ranges from 1,200 to 3,000 mm3/year. The intermediate snail hosts *Pseudosuccinea columella* and *Galba viatrix* are known to occur in the southern region of Brazil, including areas where this study was conducted, supporting the potential for fasciolosis transmission (Medeiros et al., 2014). According to federal inspection data collected between 2002 and 2011, the average annual prevalence of bovine fasciolosis in Paraná, expressed as the proportion of livers condemned at slaughter, was approximately 0.08%, with a peak of up to 9.37% recorded in 2006 (Bennema et al., 2014). In a complementary context, the analysis of buffalo livers from animals slaughtered between 2003 and 2017 indicated a prevalence of 11.9% in the state (Pritsch et al., 2019), representing a relevant cause of economic loss to the livestock industry due to liver condemnation and reduced productivity. Samples were collected from a municipal slaughterhouse in São José dos Pinhais, Paraná, which serves farms from the metropolitan region of Curitiba and neighboring municipalities, between January 2022 and December 2023 (approved by the Animals’ Ethics Committee for the Use of Animals of UFPR, CEUA number 062/2022).

### Sampling of *F. hepatica*

Adult parasites (n = 16) were collected from infected livers of beef cattle of various breeds (Nelore and its crosses) from 14 municipalities (each liver from a different municipality). The geographic coordinates of the municipalities sampled are provided in Supplementary Table S2. Two livers from Castro and Siqueira Campos, with the remaining samples obtained from Mandirituba, Ribeirão Claro, Balsa Nova, Cerro Azul, São João do Caiuá, Campina Grande do Sul, Palmeira, Japirá, São José dos Pinhais, Joaquim Távora, Tibagi, and Wenceslau Braz (Supplementary Table S1). Samples were collected at the slaughterhouse and transported to the Laboratory of Veterinary Clinical Parasitology at the Federal University of Paraná (UFPR), Curitiba, Brazil. The bile ducts were cut and squeezed to extract flukes. The parasites were then transferred to a pre-warmed saline solution (0.9% NaCl, w/v; 0.15 M). Livers were carefully sliced at 1 cm intervals, and adult parasites were added to the warm saline solution and maintained at 36 °C. Four adult *Fasciola* were collected from each liver and subsequently, the parasites were washed, carefully collected into 15 mL polypropylene Falcon tubes (Sigma-Aldrich), and preserved in 70% alcohol at –80 ºC for later use.

### Molecular profiling

#### DNA extraction, amplification, and sequencing

Genomic DNA of F. hepatica was extracted and the CO1 and NADH gene markers were amplified by PCR as previously described (Itagaki et al., 2005). PCR products were sequenced in both directions using standard methods.

#### Bioinformatic sequence analysis

Base calling and sequence accuracy procedures were performed using Gap4 software from the Staden package (Staden, 1996). Visual inspection of sequence chromatograms confirmed the presence of polymorphic sites. Evolutionary models were generated using MEGA 11 software with the lowest Bayesian information criterion (BIC) as a parameter. The HKY evolutionary model was established for *CO1* and the GTR+G evolutionary model was established for *NADH*.

A search for available published sequences was conducted using the NCBI GenBank database using the BLASTn tool. All sequences identified as from Brazil were downloaded for geographic comparisons of haplotypes, resulting in a total of 80 *NADH* sequences (RS: Arroio Grande, Camaquã, Canguçu, Herval, Ijuí, Júlio de Castilhos, Nova Prata do Iguaçu, Pejuçara, Palmeira das Missões, Santa Barbara do Sul, Santa Vitória do Palamar, Santo Cristo, São Borja, and Pelotas; PR: Curitiba; SP/São Paulo: Orto) and, 78 *CO1* sequences (RS: Arroio Grande, Camaquã, Canguçu, Herval, Ijuí, Julio de Castilhos, Nova Prata do Iguaçu, Pejuçara, Palmeira das Missões, Santa Barbara do Sul, Santo Cristo, São Borja and Pelotas; PR: Curitiba (Table S1). Subsequently, pairwise genetic distances between aligned sequences were calculated using MEGA 11 software (Hutchison & Templeton, 1999), establish the p-distance parameter (proportion of nucleotide sites). Genetic diversity parameters were estimated using DNA Sequence Polymorphism DnaSP software V6 (Rozas et al., 2017).

Phylogenetic and geographic comparisons were performed after aligning all sequences, including those of all Brazilian samples. A haplotype network was generated using the median joining method in Network software (Bandelt et al., 1999). Phylogenetic relationships within multiple *F. hepatica* samples were inferred using maximum likelihood inference on the IQ-Tree. An evolutionary model was established using MEGA 11 software, and bootstrap analysis was performed with 1,000 replicates as a statistical test.

## Results

Sixteen DNA samples from *F. hepatica* were sequenced for *CO1* and *NADH*, resulting in aligned sequences of 355 and 546 bp, respectively. We evaluated the *CO1* and *NADH* genes and identified seven haplotypes of *CO1* and four of *NADH*. To determine the intra-specific variability of *F. hepatica*, 480 *CO1* sequences were found through searches of NCBI GenBank using the BLASTn tool, with 97 sequences related to Brazil. In addition, 858 *NADH* sequences were identified, of which 96 were associated with Brazil. The *F. hepatica* population from Paraná showed higher *CO1* haplotype diversity than the complete database of samples from other parts of Brazil, including two from Paraná. Nucleotide diversity was comparable between the Brazilian and Paraná datasets, indicating low diversity (Table 1).

**Table 1:** Genetic diversity based on mitochondrial genes of *Fasciola hepatica* collected from 14 municipalities of Paraná, Brazil

**Local**	**CO1**				**NADH**			
	**Samples**	**H**	**Hd**	**π**	**Samples**	**H**	**Hd**	**π**
Paraná (new)	16	7	0,825	0,0044	16	4	0,692	0,0028
Brazil	97	12	0,624	0,0030	96	25	0,750	0,0035

* H: Number of haplotypes; Hd: Haplotype diversity and π = nucleotide diversity. Comparisons were made using NCBI GenBank sequences and the BLASTn tool.

**Figure 1:**
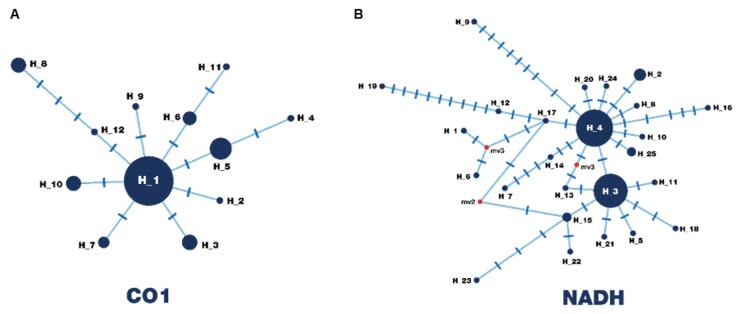
Haplotypes described in Brazil. The size of each circle is proportional to the number of samples, and each transverse line represents a mutational step.

The constructed haplotype network, which included all samples from Paraná and other parts of Brazil, revealed star-like patterns for both genes. Despite the fact that 11 haplotypes were found in *CO1* sequences from Brazil, only six haplotypes were identified in Paraná, the main one being haplotype H_5, which was found in six samples. This contrasts with Schwantes et al. (2020), who reported H_5 in only five of 79 samples, although all of them were from Paraná. In contrast, haplotype H_1, highly dominant in Schwantes’ dataset (55 samples), was observed in only three samples in the present study, confirming its occurrence in both Paraná and Rio Grande do Sul. Additionally, haplotype H_11 was found exclusively in this study. For the NADH haplotype network, of the 25 haplotypes reported in Brazil, only four were identified in Paraná: H_3 (seven samples), H_4 (four samples), H_2 (three samples), and H_11, exclusive to this study. Notably, H_3 was also widely distributed in Schwantes’ dataset (23 samples), whereas H_4, previously restricted to Rio Grande do Sul (33 samples in Schwantes et al., 2020), was here recorded for the first time in Paraná (Figure 1).

*NADH* exhibited a low genetic distance (p < 1%) among samples collected in Paraná (Table 2). The same pattern was observed when new samples from Paraná were compared to those from Brazil (Rio Grande do Sul and São Paulo). Very low values were observed for *CO1* (0–0.0084), with only Sample 10 showing values > 1% (0.0112–0.0141) (Table 3). This sample also exhibited more differences involving *NADH*, but was still < 1% (0–0.0092) for this parameter.

**Table 2:** Analysis of genetic distances (p-distance) between *NADH* haplotypes of *Fasciola hepatica* collected from infected livers of cattle in Paraná, Brazil

															

**Haplotype**	**NADH_1**	**NADH_2**	**NADH_3**	**NADH_4**	**NADH_5**	**NADH_6**	**NADH_7**	**NADH_8**	**NADH_9**	**NADH_10**	**NADH_11**	**NADH_12**	**NADH_13**	**NADH_14**	**NADH_15**
NADH_1	0.0000														
NADH_2	0,0055														
NADH_3	0,0055	0,0000													
NADH_4	0,0000	0,0055	0,0055												
NADH_5	0,0037	0,0018	0,0018	0,0037											
NADH_6	0,0055	0,0000	0,0000	0,0055	0,0018										
NADH_7	0,0055	0,0000	0,0000	0,0055	0,0018	0,0000									
NADH_8	0,0055	0,0000	0,0000	0,0055	0,0018	0,0000	0,0000								
NADH_9	0,0055	0,0000	0,0000	0,0055	0,0018	0,0000	0,0000	0,0000							
NADH_10	0,0073	0,0092	0,0092	0,0073	0,0073	0,0092	0,0092	0,0092	0,0092						
NADH_11	0,0000	0,0055	0,0055	0,0000	0,0037	0,0055	0,0055	0,0055	0,0055	0,0073					
NADH_12	0,0055	0,0000	0,0000	0,0055	0,0018	0,0000	0,0000	0,0000	0,0000	0,0092	0,0055				
NADH_13	0,0055	0,0000	0,0000	0,0055	0,0018	0,0000	0,0000	0,0000	0,0000	0,0092	0,0055	0,0000			
NADH_14	0,0037	0,0018	0,0018	0,0037	0,0000	0,0018	0,0018	0,0018	0,0018	0,0073	0,0037	0,0018	0,0018		
NADH_15	0,0037	0,0018	0,0018	0,0037	0,0000	0,0018	0,0018	0,0018	0,0018	0,0073	0,0037	0,0018	0,0018	0,0000	
NADH_16	0,0037	0,0018	0,0018	0,0037	0,0000	0,0018	0,0018	0,0018	0,0018	0,0073	0,0037	0,0018	0,0018	0,0000	0,0000

**Table 3:** Analysis of genetic distances (p-distance) between *CO1* haplotypes of 16 specimens of *Fasciola hepatica* collected from infected livers of cattle in Paraná, Brazil

**Haplotype**	**1CO1**	**2CO1**	**3CO1**	**4CO1**	**5CO1**	**6CO1**	**7CO1**	**8CO1**	**9CO1**	**10CO1**	**11CO1**	**12CO1**	**13CO1**	**14CO1**	**15CO1**
1CO1	0.0000														
2CO1	0,0028														
3CO1	0,0056	0,0028													
4CO1	0,0000	0,0028	0,0056												
5CO1	0,0056	0,0028	0,0056	0,0056											
6CO1	0,0028	0,0000	0,0028	0,0028	0,0028										
7CO1	0,0056	0,0028	0,0000	0,0056	0,0056	0,0028									
8CO1	0,0056	0,0028	0,0000	0,0056	0,0056	0,0028	0,0000								
9CO1	0,0056	0,0028	0,0000	0,0056	0,0056	0,0028	0,0000	0,0000							
10CO1	0,0141	0,0112	0,0141	0,0141	0,0141	0,0112	0,0141	0,0141	0,0141						
11CO1	0,0000	0,0028	0,0056	0,0000	0,0056	0,0028	0,0056	0,0056	0,0056	0,0141					
12CO1	0,0056	0,0028	0,0000	0,0056	0,0056	0,0028	0,0000	0,0000	0,0000	0,0141	0,0056				
13CO1	0,0056	0,0028	0,0000	0,0056	0,0056	0,0028	0,0000	0,0000	0,0000	0,0141	0,0056	0,0000			
14CO1	0,0028	0,0000	0,0028	0,0028	0,0028	0,0000	0,0028	0,0028	0,0028	0,0112	0,0028	0,0028	0,0028		
15CO1	0,0056	0,0028	0,0056	0,0056	0,0056	0,0028	0,0056	0,0056	0,0056	0,0141	0,0056	0,0056	0,0056	0,0028	
16CO1	0,0084	0,0056	0,0084	0,0084	0,0084	0,0056	0,0084	0,0084	0,0084	0,0169	0,0084	0,0084	0,0084	0,0056	0,0028

By phylogenetic analysis, homogeneity of the samples in terms of *CO1* and *NADH* sequences related to the Brazilian samples was observed. The sequences collected from Paraná exhibited low nucleotide diversity, with haplotypes diverging by only one mutation (shown in the Supplementary material by a single line connecting one haplotype to another). Even distant cities, such as Mandiruba and São João do Caiuá (650 km apart), diverged only at a single position.

## Discussion

Animal fasciolosis is prevalent in Brazil, primarily in the southern states, where the appropriate temperature and humidity, free-range grazing, and intermediate hosts (*Lymnaea* spp. snails) are present (Bennema et al., 2014; Dutra et al., 2010). This study determined the intra-specific variability of *F. hepatica* collected from cattle in different areas of Paraná. The data revealed low genetic diversity and heterogeneity traits in the population, as previously reported by Beesley et al. (2017). The apparent lack of divergence among the present samples collected from distant cities in Paraná is consistent with population expansion following a recent colonization event (Hewitt, 2000).

Analyses based on CO1 and NADH revealed low levels of diversity (< 0.01) and a star-like pattern in the haplotype network (Figure 1), indicating a recent population expansion and close evolutionary relationships among isolates. The finding of only seven CO1 and four NADH haplotypes, with most samples grouped in a few frequent haplotypes, demonstrates limited variability and high homogeneity among the parasites studied. Even samples from geographically distant municipalities (e.g., Mandirituba and São João do Caiuá, 650 km apart) showed minimal divergence, differing by only a single mutation. This uniformity suggests that *F. hepatica* populations in Paraná share a common and stable origin, with restricted gene flow or limited introduction of new variants.

When compared with reference sequences from Rio Grande do Sul and São Paulo, Paraná isolates displayed genetic distances below 1%. Such uniformity across southern Brazil may be explained by historical introductions of *F. hepatica* through livestock colonization, followed by expansion from a reduced founding stock (Bozorgomid et al., 2020). These results are also consistent with the findings of Schwantes et al. (2020), who analyzed *F. hepatica* from Rio Grande do Sul (n = 69) and Paraná (n = 10) using CO1 and NADH markers. Their study reported similarly low divergence and lack of geographical structure, supported by FST and FCT values, which agrees with the genetic homogeneity observed in our dataset. This founder effect is reinforced by the predominance of few haplotypes and low nucleotide diversity, a scenario expected when populations arise from a small number of individuals (Hewitt, 2000). The restricted cattle trade and relatively closed management systems in Paraná, together with the widespread presence of common intermediate hosts (e.g., *Pseudosuccinea columella*, *Galba viatrix*), may also contribute to the observed genetic homogeneity (Medeiros et al., 2014).

From a methodological perspective, we chose relatively short fragments of two mitochondrial genes, CO1 (355 bp) and NADH (546 bp), due to their broad use in previous studies, reliable amplification from variable-quality field samples, and availability of reference sequences in GenBank. While this approach yielded robust results, it is important to acknowledge that short fragments may underestimate variability. Cabrera et al. (2024) highlighted that longer or full-length sequences could increase resolution, and future studies should therefore incorporate extended mitochondrial fragments and additional nuclear markers to better characterize population structure.

The genetic homogeneity observed here has practical implications: on one hand, it may facilitate the development of targeted diagnostic tools, vaccines, and anthelmintics adapted to local variants; on the other, low variability increases the risk of rapid dissemination of drug-resistant alleles, as adaptive mutations can quickly spread in homogeneous populations. This dual scenario highlights the importance of continuous molecular surveillance to anticipate resistance and guide effective control strategies.

In summary, our results demonstrate that *F. hepatica* populations infecting cattle in Paraná exhibit low genetic diversity and strong homogeneity, consistent with a recent colonization and limited gene flow. These findings are in line with previous studies in Brazil and South America and provide valuable information for the epidemiological monitoring and control of fasciolosis in the region.

## Conclusion

This study expands the understanding of genetic variation in *F. hepatica* from Paraná by analyzing a larger number of field samples and characterizing intraspecific variability using mitochondrial NADH and CO1 sequences. Our findings confirm and complement those previously reported by Schwantes et al. (2020), who analyzed samples from Rio Grande do Sul and Paraná and reported low genetic diversity and lack of geographical structure of *F. hepatica* in southern Brazil. Additionally, we identified exclusive haplotypes and recorded, for the first time in Paraná, the presence of haplotype H_4, reinforcing the importance of continuous molecular surveillance in the region.

## Data Availability

The DNA sequences generated and analyzed during this study, including CO1 and NADH markers from *Fasciola hepatica* samples collected in Paraná, Brazil, are available in the NCBI GenBank database. Accession numbers for each sequence are provided in Supplementary Table S1. Additional data, including sampling locations, raw sequence alignments, and haplotype network data, are available from the corresponding author upon reasonable request.

## Supplementary Material

Supplementary material accompanies this paper.

Table S1 Samples of *Fasciola hepatica*; their geographic location (state and city), haplotype, and NCBI GenBank accession number.

Table S2 Geographic coordinates of sampling sites of *Fasciola hepatica* in cattle from Paraná, Brazil.

This material is available as part of the online article from https://doi.org/10.1590/S1984-29612025069
