# Supplementary material for: Genetic characterization of Fasciola hepatica (Linnaeus, 1758) in cattle from Paraná, Brazil
Source: Rev Bras Parasitol Vet. 2025 Dec 5;34(4):e008325. doi: 10.1590/S1984-29612025069 (PMC12704774; doi:10.1590/S1984-29612025069)
Supplement: Table S1 [file rbpv-34-4-e008325-suppl01.pdf]

SUPPLEMENTARY MATERIAL

Table S1. Samples of *Fasciola hepatica*; their geographic location (state and city), haplotype, and NCBI GenBank accession number.

State	Number	City	GenBank CO1	GenBank NADH
Rio Grande do Sul	1	Arroio Grande	MK838617	MK838695
	2	Arroio Grande	MK838621	MK838696
	3	Arroio Grande	MK838618	MK838691
	4	Arroio Grande	MK838620	MK838692
	5	Arroio Grande	MK838614	MK838689
	6	Arroio Grande	MK838619	MK838688
	7	Arroio Grande	MK838613	MK838690
	8	Arroio Grande	MK838616	MK838693
	9	Arroio Grande		MK838694
	10	Camaquã	MK838625	MK838700
	11	Camaquã	MK838622	MK838698
	12	Camaquã	MK838626	MK838699
	13	Camaquã	MK838623	MK838697
	14	Camaquã	MK838624	
	15	Canguçu	MK838628	MK838701
	16	Canguçu	MK838627	MK838702
	17	Herval	MK838678	MK838714
	18	Herval		MK838708
	19	Herval		MK838709
	20	Herval		MK838713
	21	Herval		MK838710
	22	Herval		MK838707
	23	Herval		MK838712
	24	Ijuí	MK838663	MK838715
	25	Júlio de Castilhos	MK838669	MK838720

26	Júlio de Castilhos	MK838665	MK838716
27	Júlio de Castilhos	MK838670	MK838719
28	Júlio de Castilhos	MK838664	MK838717
29	Júlio de Castilhos	MK838668	MK838718
30	Júlio de Castilhos	MK838666	
31	Júlio de Castilhos	MK838667	
32	Nova Prata do Iguaçu	MK838676	MK838724
33	Nova Prata do Iguaçu	MK838675	MK838725
34	Nova Prata do Iguaçu	MK838677	MK838723
35	Nova Prata do Iguaçu		MK838726
36	Nova Prata do Iguaçu		MK838722
37	Nova Prata do Iguaçu		MK838721
38	Pejuçara	MK838635	MK838736
39	Pejuçara	MK838636	MK838737
40	Pejuçara	MK838640	MK838734
41	Pejuçara	MK838637	MK838735
42	Pejuçara	MK838638	MK838733
43	Pejuçara	MK838639	
44	Palmeira das Missões	MK838649	MK838731
45	Palmeira das Missões	MK838653	MK838730
46	Palmeira das Missões	MK838652	MK838728
47	Palmeira das Missões	MK838649	MK838729
48	Palmeira das Missões	MK838651	MK838732
49	Palmeira das Missões	MK838655	MK838727
50	Palmeira das Missões	MK838650	
51	Palmeira das Missões	MK838654	
52	Santa Barbara do Sul	MK838658	MK838743
53	Santa Barbara do Sul	MK838656	MK838745
54	Santa Barbara do Sul	MK838657	MK838746
55	Santa Barbara do Sul		MK838744

56	Santa Barbara do Sul		MK838747
57	Santa Barbara do Sul		MK838748
58	Santa Vitória do Palmar	MK838629	MK838752
59	Santa Vitória do Palmar	MK838630	MK838754
60	Santa Vitória do Palmar	MK838633	MK838750
61	Santa Vitória do Palmar	MK838631	MK838753
62	Santa Vitória do Palmar		MK838751
63	Santa Vitória do Palmar		MK838749
64	Santo Cristo	MK838683	MK838756
65	Santo Cristo	MK838684	MK838755
66	Santo Cristo	MK838682	
67	Santo Cristo	MK838681	
68	São Borja	MK838648	MK838765
69	São Borja	MK838647	MK838757
70	São Borja	MK838662	MK838764
71	São Borja	MK838646	MK838766
72	São Borja	MK838642	MK838758
73	São Borja	MK838644	MK838761
74	São Borja	MK838647	MK838759
75	São Borja	MK838641	MK838763
76	São Borja	MK838645	MK838762
77	São Borja	MK838643	MK838760
78	São Borja	MK838660	
79	São Borja	MK838648	
80	São Borja	MK838661	
81	São Borja	MK838659	
82	Pelotas	MK838686	MK838738
83	Pelotas	MK838685	MK838740
84	Pelotas	MK838687	MK838739
85	Pelotas		MK838742

	86	Pelotas		MK838741
**Paraná**	87	Curitiba	MK838674	MK838704
	88	Curitiba	MK838672	MK838703
	89	Curitiba	MK838671	MK838705
	90	Curitiba	MK838673	MK838706
		Curitiba	MK838679	
		Curitiba	MK838680	
**São Paulo**		Orto		MF287675
**Others**			MF696152	
			MF696150	
			MF696151	

Table S2. Geographic coordinates of sampling sites of *Fasciola hepatica* in cattle from Paraná, Brazil.

Municipality	***Latitude (S)***	***Longitude (W)***
Mandirituba	25°46′44″	49°19′33″
Ribeirão Claro	23°11′38″	49°45′28″
Balsa Nova	25°35′02″	49°38′09″
Cerro Azul	24°49′26″	49°15′39″
São João do Caiuá	22°51′07″	52°20′13″
Campina Grande do Sul	25°18′21″	49°03′18″
Palmeira	25°25′44″	50°00′21″
Castro	24°47′27″	50°00′43″
Japirá	23°17′43″	50°00′39″
Siqueira Campos	23°41′16″	49°49′51″
São José dos Pinhais	25°31′00″	49°11′00″
Joaquim Távora	23°09′00″	49°56′00″
Tibagi	24°33′00″	50°28′00″
Wenceslau Braz	23°05′00″	50°11′00″
